# Safe prolonged prophylactic use and tolerability of Ashwagandha following COVISHIELD™ vaccination over 24 weeks: a multicentre randomized double-blind placebo-controlled trial

**DOI:** 10.3389/fmed.2026.1852954

**Published:** 2026-08-20

**Authors:** Arvind Chopra, Susmita Chaudhuri, Manohar S. Gundeti, S. Sarmukaddam, Girish Tillu, Preeti Chavan-Gautam, Shruti Khanduri, Sophia Jameela, Azeem Ahmad, Amit Kumar Rai, Prasanna N. Rao, Sarvesh Kumar Singh, Santosh Y. Mudakappagol, Govind Reddy, Tanuja Nesari, Dilip Gode, K. Rajeshwar Reddy, Manjit Saluja, Sanjay Tamoli, Dattatray Dighe, B. C. Ravikumar, B. G. Vittal, Shailaja U., Rakesh, M. S. Seetharamu, Kshipra Rajoria, Giridhar Vedantam, Shashikant Dudhgaonkar, Rasika Kolhe, Swapnil Borse, U. R. Sekhar Namburi, Manish P. Deshmukh, Priyanka Bangre, Anand More, S. Rajgopala, Raju Singh, Shalini Rai, Sandeep Singh Tiwari, Sanket Jain, Amrish P. Dedge, Babita Yadav, BCS Rao, N. Srikanth, R. N. Acharya, Bhushan Patwardhan

**Affiliations:** 1 Centre for Rheumatic Diseases, Pune, India; 2 Translational Health Science and Technology Institute (THSTI), Faridabad, Haryana, India; 3 Central Council for Research in Ayurvedic Sciences, New Delhi, India; 4 CCRAS-Central Ayurveda Research Institute, Mumbai, India; 5 AYUSH Center of Excellence, Center for Complementary and Integrative Health, Interdisciplinary School of Health Sciences, Savitribai Phule Pune University, Pune, India; 6 Sri Dharmasthala Manjunatheshwara College of Ayurveda and Hospital, Hassan, Karnataka, India; 7 National Institute of Ayurveda, Jaipur, India; 8 KLE SBMK Ayurveda Mahavidyalaya, Belagavi, Shahapur, Karnataka, India; 9 All India Institute of Ayurveda, Sarita Vihar, New Delhi, India; 10 DMIHER(DU) Sawangi (Meghe), Wardha, India; 11 R. A. Podar Medical College (Ayu), Mumbai, India; 12 Target Institute of Medical Education and Research, Mumbai, India

**Keywords:** Ayurveda, COVID-19 vaccine, herbal medicine, immunogenicity, safety, withaniasomnifera

## Abstract

**Introduction:**

Ashwagandha (*Withania somnifera* Dunal) is an Ayurveda medicine that was widely used during the COVID-19 pandemic. In the context of widespread use of herbal formulations alongside COVID-19 vaccination, this study evaluated the safety, tolerability, and exploratory immunological outcomes of Ashwagandha administered following COVISHIELD™ vaccination.

**Methods:**

1,200 consenting apparently healthy participants (age 18–70 years) with a negative SARS-CoV-2 RNA assay (RT-PCR using oral and nasal swab) were randomized within 7 days of vaccine administration (primary or booster) to receive oral standardized Ashwagandha (500 mg aqueous root extract) or a matching placebo daily. Participants were monitored every 4 weeks till weeks 28 (study completion) which included comprehensive clinical and laboratory assessment, specific viral RNA and IgG antibody assays (including Wuhan and Delta strain neutralization). Statistical analyses were performed using standard methods, with a two-sided *p*-value of < 0.05 considered statistically significant.

**Results:**

Of the 1,200 randomized participants, 122 (10.2%) were assigned to the SDG (Single Dose Group), 503 (41.9%) to the DDG (Double Dose Group), and 575 (47.9%) to the BDG (Booster Dose Group); overall, 1,032 participants (86%) completed the trial. The safety and tolerability profile of Ashwagandha was comparable to placebo. Adverse events were predominantly gut related and mild, and none caused withdrawal. Following first Covishield™ injection, a persistently higher IgG antibody assay was observed in the Ashwagandha arm (not statistically significant). The cumulative incidence rate of breakthrough infections was 3.9% (95% confidence interval 3.0–5.2%) and did not differ by study intervention. The immunogenic effect of Ashwagandha could not be demonstrated, likely due to a persistently high post-vaccination immune response. In addition, approximately 70% of participants were seropositive for nucleocapsid antibodies at baseline, indicating prior SARS-CoV-2 infection.

**Conclusion:**

Prolonged use and tolerability of Ashwagandha in post-vaccinated healthy participants was shown safe but adjunct immunogenicity could not be discerned. Further research is required.

**Clinical Trial Registration:**

http://www.clinicaltrials.gov/, identifier CTRI/2021/06/034496.

## 1 Introduction

An initial peak of COVID-19 cases in September 2020 was followed by a steady decline and then a second upsurge in March 2021 in India (1). This was despite stringent and timely preventive and control measures. Comprehensive vaccination guidelines were issued on December 28, 2020 and an aggressive time bound vaccination drive begun in January 2021 (1, 2). It is prudent to note that vaccines were developed on a fast track and authorized for use on an emergency basis (1). There were several vaccine concerns, more so initially, regarding safety, effectiveness, access and availability (3, 4). Although infrequent, hematological and immune-related adverse events (AE) were alarming (5). Also, there was a rapid emergence of mutant strains (SARS-CoV-2 virus) which seemed to enhance the viral infectivity and transmissibility and seemed to restrict a generalized use with any specific vaccine (6).

The Ministry of Ayush, Government of India quickly launched several initiatives including an integrative approach to manage COVID-19 (7, 8). Several Ayurvedic drugs were recommended including Rasayana Drugs, which were known to support immune functions and promote health strategies (9–12). *Withania somnifera* (L.) Dunal, commonly known as Ashwagandha, is widely recognized in Ayurvedic practiceas a highly prescribed botanical, valued for its diverse therapeutic properties and multimodal effects (12–15). Ashwagandha was specifically encouraged for research and use during the COVID pandemic (16–18). In addition, several other studies had shown an immune-modulatory effect on innate and adaptive immunity; especially involving Th1 immune response and anti-inflammatory cytokines (19, 20). As an adjuvant, it was shown to enhance the immunogenicity of diphtheria pertussis tetanus (DPT) vaccine (21).

Regardless of the evidence, a large population in India consumed Ashwagandha and other Rasayana drugs during the pandemic (22, 23). Importantly, although considered clinically safe, there were limited studies to establish the long -term safety of Ashwagandha.

It was against this background that we planned and conducted the present study on Ashwagandha to explore and determine any additional immune-mediated benefits of Ashwagandha when administered after Covishield™ vaccination. The current study was designed to target healthy consenting volunteers across India, in a placebo-controlled trial of 28 weeks duration.

## 2 Materials and methods

### 2.1 Study design

This was a prospective, randomized, double-blind, placebo-controlled, multicentre trial conducted at seven centers across India in healthy adults vaccinated with COVISHIELD™, and the detailed methodology of the study has been published previously (24).

Volunteer and apparently healthy participants who received Covishield ™ vaccination (first or second injection in the primary schedule or booster dose), were enrolled for screening on first come-first serve basis. Those qualifying were randomized and were administered either Ashwagandha or a matching placebo and monitored as per protocol (24).

The study adhered to relevant guidelines such as ASU-GCP, ICMR guidelines for human research etc., (25–27). The protocol was approved by the ethics committee at each study site and registered with the clinical trial registry (CTRI/2021/06/034496) (28).

### 2.2 Study period and setting

The qualified participants were enrolled during the period December 2021 to April 2023 at Government-accredited COVID-19 vaccination centers by the site principal investigators (PI) from Ayurveda medical institutions and Universities in New Delhi, Jaipur, Hassan, Belagavi, Nagpur, Mumbai, and Pune. The clinical phase was completed by October 2023. Each participant completed a 28-weeks study duration which included an initial 24-weeks phase of Ashwagandha administration and a final followup without intervention at 28th weeks.

### 2.3 Objectives and outcomes

The study outcomes included evaluation of the safety and tolerability of prolonged Ashwagandha administration in vaccinated adults, assessed through clinical examination, adverse event reporting, laboratory investigations, and treatment adherence over the 28-weeks study duration. Immunogenicity was evaluated by quantifying SARS-CoV-2 spike (S1) and receptor-binding domain (RBD)-specific IgG antibody titres at baseline and scheduled follow-up visits, along with neutralizing antibody responses measured using the surrogate virus neutralization test (sVNT) and serum neutralization test (SNT) against the Wuhan strain and Delta variant. Other outcomes included the sustenance of immune response, the incidence of SARS-CoV-2 breakthrough infections confirmed by RT-PCR, seroconversion rates following each vaccine dose, and changes in quality of life assessed using WHOQOL-BREF and a validated Health-Related Behavior Habit Fitness questionnaire. Details of eligibility criteria, randomization procedures, and study design are already reported (24).

### 2.4 Selection criteria

A comprehensive description of inclusion and exclusion criteria was published (24). These included participants of either sex, aged between 18 and 65 years, and testing negative for SARS-CoV-2 virus (nasal and oral swab using specific reverse transcriptase polymerase chain reaction/RT PCR technique). Any kind of co-morbidity known to impair immune system or a history of hypersensitivity to Ashwagandha was excluded. Recent use of Ashwagandha or other herbal formulations or other traditional or alternative therapies meant for COVID prophylaxis or immunity boost were excluded. Participants were screened through medical history, physical examination, and baseline laboratory investigations. The exclusion criterion relating to “known co-morbidity or acute illness” referred to clinically significant conditions that, in the investigator's judgment, could affect immune function, vaccine response, participant safety, or interpretation of study outcomes.

### 2.5 Interventions

Participants were randomized to receive Ashwagandha (study drug) or a matching placebo in a dose of 1 tab daily in the morning on an empty stomach with a glass of water. After 24 weeks of drug administration, participants were followed for a further observation period of 4 weeks. Each Ashwagandha tablet contained 500 mg of a standardized aqueous extract equivalent to 4 grams of Ashwagandha root powder.

The Covishield™ vaccination was administered according to the Government regulations and instructions provided by the manufacturer (29, 30). The primary vaccination was administered in a 2-injection dose (intramuscular) schedule with an interval of 12–14 weeks. A single booster injection dose was repeated after about 6 months of the primary vaccination.

Study medications were manufactured and procured from a government endorsed GMP-certified pharmacy for Ayurvedic drugs (Supplementary file).

### 2.6 Randomization and blinding

The vaccinated participants were randomly assigned in a 1:1 ratio to receive either Ashwagandha or a matching placebo using a centrally generated standard computer-based randomization sequence; allocation concealment by using block randomization. The investigational product and its matching placebo were identical in shape, color, appearance, and packaging, and were dispensed in similarly labeled containers. Each container was assigned a unique enrolment number generated centrally by an independent biostatistician, thereby ensuring allocation concealment and maintaining blinding of participants, investigators, and study personnel throughout the trial.

### 2.7 Protocol amendment

A large proportion of India's population was vaccinated in 2021–22. By the end of Jan 2023, the study enrolment was substantially reduced because of lesser number of volunteer participants available after the first vaccine injection of the primary schedule according to the initial protocol. After considerable discussion and expert opinion, the protocol was amended and approved by the EC to additionally facilitate screening of participants immediately after the second injection of the primary vaccination schedule or the booster dose and enroll the qualified on first come first serve basis. This led to 3 subset study groups- after single/first injection group (SDG), after double/primary vaccination (DDG) and after booster (BDG).

### 2.8 Statistical methods and reporting

There was no previous data on the current subject for sample size calculations. However, based on relevant literature review and consensus (SS, AC), a margin of 5–7% in the effect size between the two current study intervention groups with respect to any of the specific SARS-CoV-2 antibodies (IgG) assay and favoring Ashwagandha was accepted for calculating the sample size with 80% statistical power and an alpha of 0.05; 70–80% participants were considered to show a successful immune response (elevated serum S1 and RBD assay) at weeks 4–12 following vaccination. Finally, a sample size of 1,200 participants were approved; this included a 10% dropout rate.

Safety and tolerability analyses included all randomized participants who received at least one dose of the study intervention (ITT/safety population). Immunogenicity analyses were performed on the per-protocol population comprising participants who completed the scheduled follow-up and immunogenicity assessments in accordance with the protocol. The results of immunogenicity endpoints were presented as per-protocol analysis.

Standard statistical software (including SPSS Statistics Version 26.0.) was used for analysis by a senior biostatistician (SS).

Immunogenicity assays (S1, RBD and NP) were analyzed using geometric means titres (GMTs and log-normal distribution). Proportions were compared for the virus neutralization test (SNT and sVNT). Fisher's exact test was used for analysis of number of participants with breakthrough COVID-19 infections. Chi-square was used for categorical outcomes to analysesafety data. Statistical significance was *p* < 0.05, two-tailed and 95% confidence intervals (CI) were shown. This report was compliant to the CONSORT guidelines (31).

## 3 Results

**Figure 1:**
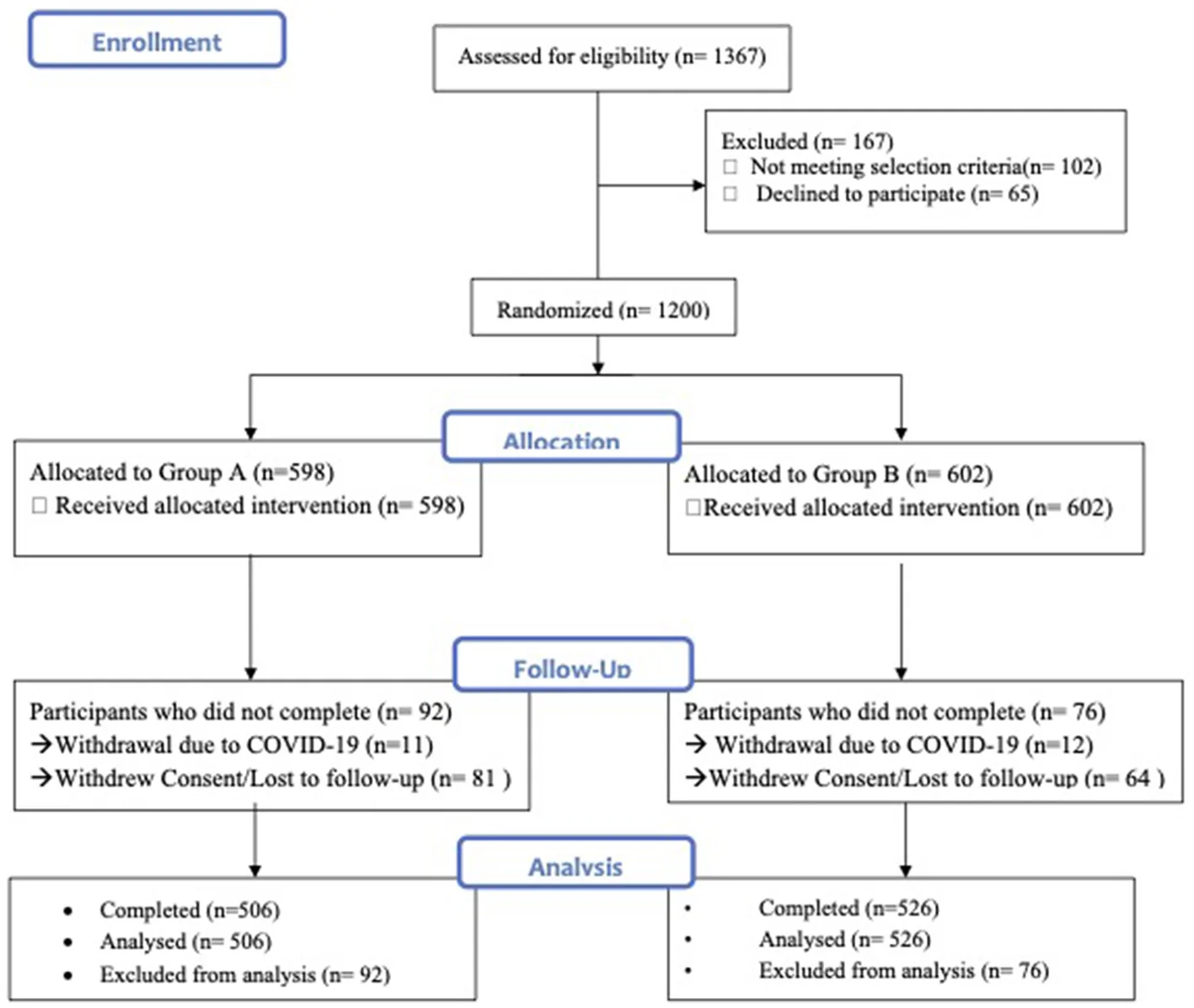
CONSORT Flow diagram of participant enrollment, allocation, follow-up, and analysis.

The CONSORT study flow including allocation of study participants is shown in Figure 1.

A total of 1,200 participants were randomized: 122 (10.2%) to the SDG, 503 (41.9%) to the DDG, and 575 (47.9%) to the BDG. Overall, 1,032 participants (86.0%) completed the trial, including 104 in the SDG, 384 in the DDG, and 544 in the BDG.

A total of 92 participants (15.38%) in the Ashwagandha group and 76 (12.62%) participants in the placebo group were withdrawn with no statistically significant difference between groups (χ^2^ test, *p* = 0.183).

Table 1 presents the baseline demographic and selected clinical characteristics of the study participants. The distribution of these characteristics was comparable between the study groups. Participants were predominantly young adults of both sexes, most of whom were students or salaried employees; none were employed in medical or healthcare settings. A history of prior symptomatic COVID-19 was reported by 63 participants (5.3%), and among these individuals, 68.2% were subsequently identified as seropositive for SARS-CoV-2 nucleocapsid antibodies, consistent with previous infection.

**Table 1:** Demographic and clinical characteristics at baseline.

Variable	Single dose (*n* = 122)			Double dose (*n* = 502)			Booster dose (*n* = 570)			Combined (*n* = 1194)		
	A (*n* = 59)	B (*n* = 63)	*P*	A (*n* = 251)	B (*n* = 251)	*p*	A (*n* = 282)	B (*n* = 288)	*p*	A (*n* = 592)	B (*n* = 602)	*P*
Age (years) mean (SD)	28.5 (8.1)	26.5 (7.9)	0.15	28.9 (7.9)	28.0(8.2)	0.17	30.2 (12.0)	30.8 (11.7)	0.44	29.5 (10.1)	29.2 (10.1)	0.40
Height (cm) mean (SD)	157.3 (10.3)	161.2 (9.6)	0.03	161.4 (10.7)	161.8 (10.6)	0.98	162.7 (9.2)	163.5 (22.5	0.82	161.6 (10.1)	162.5 (17.3)	0.64
Weight (Kg) mean (SD)	61.2 (10.4)	60.9 (9.9)	0.61	63.8 (12.9)	61.8 (10.8)	0.04	63.0 (13.2)	62.5 (13.1)	0.42	63.2 (12.8)	62.0 (11.8)	0.05
Body mass index mean (SD)	24.8 (4.1)	23.5 (3.4)	0.07	24.6 (5.1)	23.8 (4.4)	0.06	23.8 (4.6)	23.6 (4.4)	0.62	24.2 (4.8)	23.6 (4.3)	0.03
Male *n* (%)	38 (64.41)	40 (63.49)	0.15	169 (67.3)	175 (69.3)	0.33	131 (46.4)	128 (44.44)	0.63	339 (57.27)	343 (56.94)	0.89
Students *n* (%)	15 (25.42)	23 (36.51)	0.07	88 (35.1)	99 (39.4)	0.31	127 (45)	124 (43.06)	0.63	204 (34.55)	211 (35.08)	0.83
Education (12^th^ pass or more), *n* (%)	32 (54.24)	34 (53.97)	0.14	155 (61.8)	158 (62.9)	0.78	249 (88.3)	239 (83.01)	0.07	436 (73.73)	431 (71.63)	0.43
Married, *n* (%)	24 (40.68)	20 (31.75)	0.08	120 (47.8)	98 (39.0)	0.02	93 (33.0)	103 (35.76)	0.48	237 (40.10)	221 (36.71)	0.24
Interval between vaccination to randomization mean (SD)	3.29 (1.81)	3.25 (1.79)	0.93	3.33 (1.95)	3.42 (1.97)	0.61	2.74 (1.48)	2.82 (1.54)	0.66	3.04 (1.75)	3.11 (1.78)	0.50
Seropositive nucleocapsid protein present, *n* (%)	40 (74.1)	37 (66.1)	0.83	156 (72.6)	177 (73.8)	0.08	224 (79.2)	253 (87.9)	0.005	420 (70.94)	467 (77.57)	0.01

*n*, Number of participants; A, Ashwagandha; B, Placebo; *p*, Mann Whitney/Chi-square statistic, significance *p* < 0.05, Two tailed; All participants vaccinated with COVISHIELD^TM^ vaccination. 68 samples could not be tested because of several reasons including participant withdrawal and unsuitable sample; Total study cohort sample size 1200; IgGNucleocapsid antibody tested by standard ELISA (cut off 0.2 Iu/ml).

## 4 Efficacy outcomes

### 4.1 Serum assays

Table 2 summarizes the geometric mean titres of SARS-CoV-2–specific S1, RBD, and nucleocapsid (NP) antibodies. At baseline, antibody levels were several-fold higher than the predefined seropositivity cut-off (established *a priori* through in-house standardization), with more than 95% of participants seropositive across all study groups. No statistically significant differences were observed between the Ashwagandha and placebo groups at any predefined study time point.

**Table 2:** Serum assay results of Spike1 (S1), Receptor Binding Domain (RBD) and Nucleocapsid Protein (NP) antibody in single dose sub group.

Variable	Dose	Baseline		Mean (SD) (Week12)		Mean (SD) (weeks 24)		Mean (SD) (Week 28)		p1	p2	p3	p4
		A	B	A	B	A	B	A	B				
S1	Single dose	129.44 (7.32) (*n* = 48)	173.15 (5.78) (*n* = 56)	378.91 (1.92) (*n* = 46)	395.4 (1.79) (*n* = 54)	306.49 (2.08) (*n* = 46)	331.95 (1.78) (*n* = 51)	301.65 (1.75) (*n* = 46)	308.43 (1.63) (*n* = 51)	0.39	0.92	0.91	0.97
	Double dose	317.48 (1.80) (*n* = 182)	312.28 (2.02) (*n* = 193)	372.89 (1.69) (*n* = 173)	380.28 (1.75) (*n* = 192)	295.77 (1.59) (*n* = 164)	309.32 (1.47) (*n* = 168)	305.82 (1.59) (*n* = 174)	311.41 (1.64) (*n* = 181)	0.63	0.71	0.26	0.65
	Booster dose	334.72 (1.67) (*n* = 266)	362.24 (1.58) (*n* = 273)	287.67 (1.63) (*n* = 193)	300.46 (1.58) (*n* = 197)	250.23 (1.25) (*n* = 253)	251.64 (1.18) (*n* = 255)	243.11 (1.35) (*n* = 265)	248.68 (1.29) (*n* = 265)	0.35	0.34	0.76	0.44
	Combined	299.97 (2.33) (*n* = 496)	316.78 (2.26) (*n* = 522)	331.02 (1.7) (*n* = 413)	344.09 (1.70) (*n* = 443)	270.91 (1.50) (*n* = 463)	278.94 (1.41) (*n* = 474)	269.48 (1.51) (*n* = 486)	275.92 (1.49) (*n* = 500)	0.21	0.37	0.31	0.36
RBD	Single dose	96.57 (5.91)	144.38 (4.20)	260.8 (1.88)	266.58 (1.52)	269.15 (2.17)	294.83 (1.97)	222.43 (1.69)	229.98 (1.56)	0.26	0.71	0.66	0.87
	Double dose	277.5 (1.93)	270.05 (2.19)	289.46 (2.47)	306.46 (1.67)	302.59 (1.68)	319.57 (1.59)	261.38 (1.45)	262.01 (1.54)	0.98	0.98	0.39	0.81
	Booster dose	350.23 (2.02)	392.7 (1.58)	313.41 (1.88)	341.18 (1.53)	246.43 (1.37)	253.40 (1.19)	254.24 (1.43)	257.29 (1.34)	0.13	0.27	0.79	0.88
	Combined	284.34 (2.52)	306.99 (2.25)	296.93 (2.13)	316.02 (1.59)	267.36 (1.59)	279.63 (1.48)	253.48 (1.47)	255.90 (1.44)	0.35	0.82	0.32	0.85
NP	Single dose	0.53 (7.96)	0.43 (6.64)	0.66 (4.62)	0.87 (5.12)	0.68 (5.57)	0.55 (5.22)	1.03 (4.04)	0.92 (4.28)	0.39	0.45	0.51	0.58
	Double dose	0.69 (5.00)	0.68 (5.69)	1.22 (4.99)	0.96 (5.39)	1.03 (3.49)	0.99 (3.95)	1.36 (2.89)	1.23 (3.15)	0.61	0.22	0.92	0.47
	Booster dose	0.72 (4.19)	0.88 (3.50)	1.36 (3.26)	1.58 (2.60)	1.19 (3.14)	1.29 (2.76)	1.29 (2.94)	1.38 (2.48)	0.25	0.18	0.73	0.77
	Combined	0.69 (4.80)	0.74 (4.66)	1.20 (4.17)	1.18 (4.14)	1.07 (3.52)	1.07 (3.51)	1.28 (3.03)	1.26 (2.92)	0.45	0.85	0.95	0.69
SVNT	Single dose			89.4 (17.3)	85.9 (26.5)	87.0 (23.8)	85.8 (26.3)	86.8 (26.0)	87.0 (26.4)		0.74	0.63	0.19
	Double dose			91.7 (13.1)	93.0 (9.2)	92.6 (15.1)	93.7 (11.6)	93.4 (13.1)	93.0 (12.6)		0.54	0.85	0.27
	Booster dose			93.6 (12.3)	93.9 (11.5)	94.0 (13.8)	95.5 (7.5)	94.3 (9.9)	94.1 (12.1)		0.75	0.17	0.89
	Combined			92.3 (13.3)	92.6 (13.6)	92.8 (15.6)	93.8 (11.9)	93.2 (15.5)	93.1 (12.6)		0.39	0.40	0.68

*n*, Number of participants; *A*, Ashwagandha; *B*, Placebo; *p*, Mann Whitney/Chi-square, significance *p* < 0.05, Two tailed; All participants vaccinated with COVISHIELD^TM^ vaccination; week, week; cut-off titers for S1 ≥ 5 IU/ml, RBD ≥ 5 IU/ml, seropositive NP, 0.2 IU/ml; geometric mean is the log value (log e); p1, p2, p3, p4 pertain to baseline, weeks 12, 24, and 28 respectively; SVNT: Surrogate virus neutralization titer.

The proportion of participants demonstrating virus-neutralizing activity was high across all study groups, exceeding 85% by both surrogate and serum neutralization assays (Table 2). These proportions were comparable between intervention arms at all assessment points, with no statistically significant differences.

Table 3 presents longitudinal changes in S1, RBD, and NP antibody levels at predefined study endpoints across vaccination subgroups. Antibody trajectories over time did not differ significantly between the Ashwagandha and placebo groups.

**Table 3:** Change (geometric mean) from baseline (week 0) in serum assay titres of Spike1 (S1), Receptor Binding Domain (RBD) and Nucleocapsid Protein (NP) antibody.

Variable	Dose	Change (0–12 weeks)		Change (0–24 weeks)		Change (0–28 weeks)		P1 (0–12)	P2 (0–24)	P3 (0–28)
		A	B	A	B	A	B			
S1	Single dose	2.79 (6.89)	2.33 (6.58)	2.46 (5.56)	1.96 (6.69)	2.30 (6.19)	1.87 (5.43)	0.53	0.24	0.45
	Double dose	1.19 (2.27)	1.23 (2.43)	0.95 (2.18)	1.00 (2.25)	0.98 (2.07)	0.97 (2.52)	0.89	0.96	0.47
	Booster dose	0.85 (1.97)	0.84 (1.96)	0.75 (1.65)	0.70 (1.58)	0.72 (1.78)	0.68 (1.67)	0.97	0.37	0.54
	Combined	1.12 (2.76)	1.12 (2.82)	0.92 (2.39)	0.88 (2.48)	0.90 (2.44)	0.86 (2.51)	0.86	0.25	0.27
RBD	Single dose	2.63 (4.81)	1.89 (3.93)	2.91 (5.00)	2.07 (4.70)	2.29 (4.64)	1.63 (4.00)	0.10	0.36	0.25
	Double dose	1.05 (2.96)	1.13 (2.39)	1.08 (2.49)	1.19 (2.54)	0.94 (1.99)	0.95 (2.45)	0.96	0.68	0.67
	Booster dose	0.89 (2.69)	0.88 (1.90)	0.70 (2.05)	0.64 (1.63)	0.73 (2.12)	0.66 (1.71)	0.82	0.23	0.22
	Combined	1.07 (3.16)	1.08 (2.44)	0.96 (2.83)	0.90 (2.52)	0.89 (2.46)	0.83 (2.35)	0.78	0.40	0.12
NP	Single dose	1.23 (7.30)	2.08 (6.50)	1.38 (7.19)	1.16 (4.62)	1.90 (6.53)	2.18 (5.89)	0.14	0.88	0.57
	Double dose	1.71 (5.79)	1.43 (6.78)	1.55 (5.83)	1.56 (5.71)	1.89 (5.66)	1.74 (5.60)	0.16	0.90	0.51
	Booster dose	1.83 (5.05)	1.74 (4.28)	1.68 (5.16)	1.49 (4.46)	1.80 (4.87)	1.61 (4.28)	0.75	0.87	0.73
	Combined	1.70 (5.60)	1.63 (5.58)	1.60 (5.57)	1.47 (4.91)	1.84 (5.3)	1.71 (4.91)	0.88	0.99	0.69

*n*, Number of participants; *A*, Ashwagandha; *B*, Placebo; *p*, Mann Whitney/Chi-square, significance *p* < 0.05, Two tailed; All participants vaccinated with COVISHIELD ^TM^ vaccination; weekk, week; cut-off titers for S1 ≥ 5 IU/ml, RBD ≥ 5 IU/ml, seropositive NP = 0.2 IU/ml; geometric mean is the log value (log e), p1, p2, p3 pertain to mean change from baseline to weeks 12, 24 and 28 respectively.

Modest numerical differences favoring the Ashwagandha arm were observed in the single-dose group (SDG; participants enrolled following the first vaccine dose). In this subgroup, serum S1 and RBD antibody levels were numerically higher in the Ashwagandha arm than in the placebo arm across the study time points (Table 3); however, none of these between-group differences reached statistical significance. Similarly, the proportion of participants demonstrating surrogate virus neutralization activity was numerically higher in the Ashwagandha arm than in the placebo arm (89.4 vs. 85.9%), although the difference was not statistically significant. Comparable numerical differences were not observed in the double-dose (DDG) or booster-dose (BDG) groups.

At randomization, 74.25% of participants were seropositive for SARS-CoV-2 nucleocapsid antibodies, with comparable proportions in the Ashwagandha (70.94%) and placebo (77.57%) groups (*p* = 0.50) (Table 2). Only marginal increases in nucleocapsid antibody positivity were observed over time (Table 3), with seropositivity exceeding 95% by weeks 28 across all vaccination subgroups, irrespective of study intervention. These increases were most likely attributable to natural exposure, breakthrough infections, or community transmission during the study period.

However, there were some observational trends in the specific antibody response to favor the Ashwagandha arm in the SDG sub-group (enrolment following first vaccine injection). The serum S1 and RBD assays in the Ashwagandha study arm were 20–40% higher at all study time points (Table 3). The proportion showing surrogate virus neutralization assay was 89.4% in the Ashwagandha arm vs. 85.9% in the placebo (not significant). This kind of a persistent humoral immune response was not seen in either the DDG or BDG Groups.

### 4.2 Breakthrough infections

There were 47 participants with breakthrough infections (11 symptomatic, 36 asymptomatic) with a cumulative incidence rate of 3.9% (95% CI: 3.0–5.2%) in the current study population (Table 4). There was no significant difference in the proportion of COVID-19 cases between the two study intervention (95% CI: −1.0–3.5%) in the total study sample; this was also seen in each of the study groups (Table 4).

**Table 4:** Breakthrough infections.

Sub-group	Group A	Group B	95% CI of the difference	*P* ^*^
Single dose	5 (8.5%, 3.7–18.4%)	2 (3.2%, 0.9–10.9%)	−3.8–15.4	0.26
Double dose	16 (6.3%, 3.9–10.1%	11 (4.4%, 2.5–7.7%0	−2.11–6.1	0.42
Booster	6 (2.1%, 0–4.5%)	7 (2.4%, 1.2–4.9%)	−3.1–2.4	1
Study cohort	27 (4.5%, 3.1–6.5%)	20 (3.3%, 2.2–5.1%)	−1–3.5	0.30

^*^Fisher Exact Chi-square statistic, two tailed; 59, 252, 287 and 598 participants randomized to Group A in the Single Dose, Double Dose, Booster, and Total Study Cohort respectively; corresponding 63, 251, 288 and 602 participants randomized to Group B; Symptomatic COVID-19 infection diagnosed by trained physician and confirmed by specific RT-PCR assay on nose/throat swab.

All symptomatic COVID-19 participants made uncomplicated recovery within 5–7 days.

### 4.3 Safety

A total of 337 adverse events (AEs) were reported in the Ashwagandha group and 309 in the placebo group (Table 5). The majority of AEs were mild to moderate in severity, self-limiting, and resolved within 1 or 2 days, typically without the need for medical intervention.

**Table 5:** Incidence of Adverse Events (AE) in vaccinated participants who were administered Aswagandha/placebo.

Adverse event	Group A (*n* = 598)				Group B (*n* = 602)			
	Number		% (events)	Exact 95% CI for % [as recommended by Wilson]	Number		% (events)	Exact 95% CI for % [as recommended by Wilson]
	Participants	Events			Participants	Events		
Constipation	8	12	2.0%	1.2–3.5%	4	8	1.3%	0.7–2.6%
Hyperacidity	9	16	2.7%	1.7–4.3%	10	18	2.7%	1.7–4.3%
Acute abdomen	9	10	1.7%	0.9–3.1%	8	8	1.3%	0.7–2.6%
Diarrhea	9	13	2.2%	1.2–3.7%	6	11	1.8%	1.0–3.2%
Dyspepsia	5	5	0.8%	0.4–1.9%	5	7	1.2%	0.6–2.4%
Fever (non-specific)	21	32	5.4%	3.8–7.5%	9	19	3.2%	2.0–4.9%
RT PCR positive symptomatic Covid−19	11	11	1.8%	1.2–3.5%	11	11	1.8%	0.9–3.1%
RT-PCR positive asymptomatic Covid-19	16	16	2.7%	1.5–4.1%	9	9	1.5%	0.9–3.1%
Neck back pain	8	14	2.3%	1.4–3.9%	6	10	1.7%	0.9–3.1%
Soft tissue pain	4	6	1.0%	0.5–2.2%	11	16	2.7%	1.7–4.3%
Arthralgia	4	5	0.8%	0.4–1.9%	9	14	2.3%	1.4–3.9%
Allergic dermatitis	5	7	1.2%	0.6–2.4%	7	7	1.2%	0.6–2.4%
Infective dermatitis	4	6	1.0%	0.5–2.2%	2	2	0.3%	0.1–1.2%
URI	65	100	16.7%	13.9–19.9%	64	96	15.9%	13.2–19.1%
Sleep abnormality	4	9	1.5%	0.8–2.8%	5	11	1.8%	1.0–3.2%
Giddiness	8	11	1.8%	1.0–3.2%	4	4	0.7%	0.3–1.7%
Menstrual abnormality	12	12	2.0%	1.2–3.5%	14	20	3.3%	2.2–5.1%
Abnormal LFT	6	12	2.0%	1.2–3.5%	3	4	0.7%	0.3–1.7%
Injury	9	9	1.5%	0.8–2.8%	8	9	1.5%	0.8–2.8%
Headache	20	29	4.8%	3.4–6.9%	13	18	3.0%	1.9–19.1%
Lethargy	1	2	0.3%	0.1–1.2%	5	7	1.2%	0.6–2.4%

This table displays the number of participants and the percentage of adverse events (AEs) occurring with an incidence rate of 1% or greater in intervention groups A and B of the study cohort (*n* = 1,200). The table includes the incidence rate of each AE along with the corresponding 95% confidence intervals, providing a detailed view of AE frequency and variability within the study groups. n: Number of participants; A, Ashwagandha; B, Placebo; *p*, Fisher's Exact Test, significance *p* < 0.05, Two tailed; All participants vaccinated with COVISHIELD ^TM^ vaccination; URI, Upper Respiratory Infection; LFT, Liver Function Test.

Eight serious adverse events (SAEs) were reported during the study, none of which were considered related to the study intervention. Six SAEs occurred in the Ashwagandha group [acute appendicitis (*n* = 1), acute colitis (*n* = 1), non-specific acute abdominal pain (*n* = 1), non-specific acute chest pain (*n* = 1), and road traffic accidents (*n* = 2)], and two SAEs occurred in the placebo group [acute gastroenteritis (*n* = 1) and acute acid-peptic disorder (*n* = 1)]. All SAEs were evaluated and managed by specialist physicians in hospital settings, and all affected participants recovered fully without complications.

In 9 participants (Ashwagandha 7, placebo 2), the AE were reported as having a possible/probable causal relationship with the study intervention. In the Ashwagandha group, these events were assessed as having a possible causal relationship with the study intervention and were clinically classified as mild to moderate, including unexplained changes in body weight, oral mucosal ulcers, acid-peptic symptoms, excessive sleep, and giddiness. In the placebo group, unexplained weight gain and excessive sleep were reported.

In several participants with mild to moderate AE, irrespective of the study intervention, the study medication was stopped for 1–3 days. These were mostly managed with Ayurvedic measures and rarely required symptomatic allopathic drugs.

The results of laboratory investigations are shown in Supplementary Table S1. All laboratory parameters tested according to the study protocol, including routine hematology, blood sugar, liver and renal function, and lipids remained within the reference range at all pre-determined study time points.

The study medications were well-tolerated. 168 (14%) participants were withdrawn from the study due to various reasons but not because of any drug related adverse event (AE) (Figure 1); 18 (14.7%) in SDG, 119 (23.7%) in DDG and 31 (5.4%) in the BDG. There were no deaths reported in the study. The AE in the Ashwagandha group were comparable to the placebo at all study time points.

It is prudent to add none of the AE recorded in the study were considered due to vaccination (Covishield™). Prior to baseline enrolment, several participants recalled local discomfort and mild febrile aches and pains that resolved within 1 day.

Quality of Life Outcomes: there were no statistically significant differences between the study intervention groups for WHO Quality of Life (QOL) Bref and HR-BHF questionnaire scores (Supplementary Table S3).

## 5 Discussion

In this randomized, placebo-controlled trial involving 1,200 adults vaccinated with Covishield™, prolonged administration of Ashwagandha was evaluated over 28 weeks for safety, tolerability, and immunological outcomes. Ashwagandha was well tolerated, with high study completion (86%) and no serious adverse events attributable to the intervention.

An early and persistent robust humoral immune response following Covid vaccination (Covishield™) was observed in both the study arms, which was unanticipated and much more than what was reported (30, 32). The cumulative incidence of breakthrough SARS-CoV-2 infection among vaccinated participants in the present study was 3.9% (95% CI 3.0%−5.2%). This incidence is lower than that reported among vaccinated cohorts in Indian observational studies conducted during the second wave, particularly in high-exposure settings (33, 34). Laboratory parameters, including hematological, hepatic, and renal function tests, remained within normal ranges, (Table 6, Supplementary material, Supplementary Table S1). Immunogenicity analyses indicated a positive trend toward numerically higher antibody responses when the intervention was initiated following the first vaccine dose; however, a definitive immunogenic effect could not be established.

**Table 6:** Effect of Ashwagandha administration in vaccinated individuals on Laboratory parameters (CBC, LFT, RFT).

Variables	Dose	Baseline		Mean (SD) (weak 12)		Mean (SD) (weak 24)		p1	p2	p3
No of participants = *n*		A	B	A	B	A	B			
(*S*-single dose)		*S* = 48	*S* = 56	*S* = 46	*S* = 53	*S* = 47	*S* = 51			
*D* (double dose)		*D* = 185	*D* = 197	*D* = 172	*D* = 181	*D* = 161	*D* = 169			
*B* (booster dose)		*B* = 271	*B* = 273	*B* = 189	*B* = 192	*B* = 246	*B* = 258			
Hb (g/dl)	First dose	14.34 (2.03)	14.58 (1.84)	14.19 (1.77)	13.91 (1.60)	13.87 (1.96)	14.09 (1.69)	0.714	0.714	0.324
	Second dose	14.31 (1.91)	14.26 (2.04)	14.09 (1.85)	14.11 (2.02)	13.93 (1.97)	13.96 (1.99)	0.934	0.316	0.882
	Booster dose	14.09 (1.98)	13.78 (1.98)	13.62 (1.83)	13.41 (1.85)	13.89 (1.84)	13.60 (1.81)	0.802	0.701	0.593
Total WBC (x10^∧^3/μL)	First dose	7.60 (2.04)	7.79 (2.11)	6.72 (1.44)	6.90 (1.76)	6.82 (1.69)	7.24 (1.54)	0.556	0.156	0.381
	Second dose	7.03 (1.99)	7.09 (2.06)	6.70 (1.67)	6.74 (1.71)	6.99 (1.75)	6.73 (1.52)	0.639	0.543	0.199
	Booster dose	7.36 (1.88)	7.21 (1.77)	7.09 (1.58)	7.02 (1.92)	7.26 (1.71)	7.40 (1.78)	0.760	0.123	0.324
Neutrophils (%)	First dose	57.33 (9.95)	58.52 (9.61)	55.03 (8.58)	56.08 (8.73)	57.72 (9.83)	58.60 (10.35)	0.843	0.911	0.822
	Second dose	59.08 (8.46)	58.84 (9.58)	59.58 (7.86)	58.82 (8.20)	60.82 (9.80)	61.14 (9.91)	0.126	0.461	0.571
	Booster dose	59.30 (7.80)	59.15 (8.48)	57.69 (7.84)	56.99 (8.33)	58.00 (9.66)	58.78 (8.69)	0.589	0.599	0.152
Lymphocyte (%)	First dose	32.60 (9.10)	31.40 (8.34)	34.53 (6.30)	34.24 (6.18)	34.08 (7.53)	31.06 (7.80)	0.401	0.489	0.821
	Second dose	31.26 (7.15)	31.77 (8.35)	31.44 (6.83)	32.36 (7.35)	30.32 (7.90)	30.62 (7.94)	0.064	0.298	0.841
	Booster dose	31.24 (7.42)	31.54 (7.56)	32.63 (7.57)	33.81 (7.63)	32.65 (8.42)	32.15 (7.16)	0.951	0.823	0.017
Basophils (%)	First dose	0.52 (0.71)	0.49 (0.59)	0.37 (0.52)	0.49 (0.62)	0.38 (0.56)	0.51 (0.59)	0.219	0.198	0.368
	Second dose	0.29 (0.64)	0.27 (0.47)	0.26 (0.75)	0.25 (0.49)	0.20 (0.35)	0.24 (0.64)	0.527	0.710	0.180
	Booster dose	2.14 (2.34)	0.30 (0.43)	0.16 (0.33)	0.17 (0.31)	0.28 (0.45)	0.30 (0.44)	<0.001	0.914	0.766
Eosinophil (%)	First dose	3.82 (2.85)	4.13 (3.20)	5.11 (5.62)	4.26 (2.56)	4.38 (2.98)	3.56 (1.93)	0.582	0.273	0.022
	Second dose	3.66 (2.89)	3.42 (3.11)	3.58 (2.65)	3.25 (2.30)	3.65 (3.24)	3.65 (3.31)	0.943	0.147	0.727
	Booster dose	4.61 (2.97)	2.96 (3.24)	3.47 (3.07)	3.35 (3.04)	3.39 (3.20)	3.31 (3.21)	0.003	0.521	0.430
ESR mm/hr	First dose	16.60 (13.99)	20.55 (14.08)	17.14 (12.62)	17.42 (6.74)	13.89 (8.20)	18.98 (10.56)	0.550	<0.001	0.921
	Second dose	16.18 (14.40)	15.58 (14.42)	12.76 (10.03)	13.76 (11.40)	15.51 (17.07)	12.48 (10.18)	0.609	0.269	0.014
	Booster dose	13.40 (13.77)	15.05 (13.10)	14.65 (13.54)	15.46 (12.51)	15.65 (14.61)	19.32 (17.60)	0.949	0.943	0.107
Platelet (x10^∧^3/μL)	First dose	281.15 (76.06)	279.93 (81.41)	265.74 (67.41)	289.77 (97.72)	279.26 (72.31)	277.47 (76.92)	0.663	0.036	0.454
	Second dose	250.91 (85.91)	256.54 (93.43)	242.14 (81.89)	250.66 (74.47)	259.16 (76.75)	261.46 (71.11)	0.486	0.336	0.461
	Booster dose	268.71 (81.48)	272.55 (81.69)	279.77 (81.62)	273.53 (80.18)	281.90 (71.87)	279.70 (75.86)	0.315	0.824	0.560
SGOT (U/L)	First dose	30.14 (34.52)	27.52 (22.66)	33.90 (38.32)	27.58 (15.14)	30.69 (27.54)	27.67 (10.58)	0.736	0.363	0.152
	Second dose	25.42 (10.38)	25.48 (13.14)	25.16 (9.36)	25.61 (10.25)	24.08 (7.86)	27.68 (13.61)	0.642	0.531	<0.001
	Booster dose	23.09 (10.21)	23.05 (10.51)	23.58 (20.34)	22.84 (8.69)	22.24 (7.99)	22.70 (13.48)	0.716	0.352	0.088
SGPT (U/L)	First dose	29.27 (28.39)	31.27 (34.41)	32.03 (21.39)	29.82 (25.77)	33.50 (23.62)	30.18 (16.88)	0.591	0.645	0.013
	Second dose	26.73 (17.98)	25.97 (17.50)	25.36 (14.74)	26.59 (16.11)	25.74 (13.49)	29.40 (17.67)	0.531	0.337	0.007
	Booster dose	24.46 (16.41)	22.78 (15.37)	24.33 (24.30)	22.34 (15.50)	23.54 (15.82)	23.29 (19.29)	0.119	0.104	0.840
Bilirubin (mg/dL)	First dose	0.78 (0.44)	0.75 (0.38)	0.70 (0.33)	0.75 (0.40)	0.65 (0.27)	0.72 (0.38)	0.559	0.808	0.081
	Second dose	0.77 (0.38)	0.81 (0.56)	0.81 (0.42)	0.84 (0.53)	0.83 (0.41)	0.82 (0.46)	0.014	0.027	0.154
	Booster dose	0.81 (0.50)	0.78 (0.45)	0.77 (0.48)	0.76 (0.39)	0.71 (0.38)	0.71 (0.43)	0.770	0.370	0.329
Alkaline phosphate (U/L)	First dose	87.45 (30.86)	82.61 (27.08)	81.07 (30.14)	72.31 (21.95)	80.27 (26.22)	72.57 (25.40)	0.367	0.035	0.975
	Second dose	80.93 (25.63)	83.24 (26.17)	79.56 (22.94)	80.94 (28.65)	76.50 (23.79)	77.80 (26.23)	0.752	0.071	0.320
	Booster dose	77.11 (22.32)	78.01 (21.36)	75.65 (20.15)	75.10 (20.47)	76.26 (21.95)	76.89 (20.67)	0.636	0.315	0.899
Albumin (g/dL)	First dose	4.62 (0.60)	4.61 (0.30)	4.53 (0.32)	4.53 (0.28)	4.54 (0.32)	4.47 (0.36)	0.119	0.465	0.843
	Second dose	4.51 (0.55)	4.53 (0.38)	4.55 (0.42)	4.48 (0.33)	4.57 (0.375)	4.53 (0.38)	0.022	0.455	0.660
	Booster dose	4.64 (0.33)	4.63 (0.33)	4.60 (0.39)	4.60 (0.46)	4.70 (0.32)	4.68 (0.33)	0.930	0.303	0.573
Globulin (g/dL)	First dose	3.15 (0.53)	3.10 (0.58)	3.15 (0.53)	3.15 (0.52)	3.13 (0.45)	3.08 (0.42)	0.298	0.826	0.859
	Second dose	3.21 (2.71)	2.99 (0.39)	2.97 (0.48)	2.97 (0.46)	2.89 (0.59)	2.98 (0.61)	0.033	0.700	0.733
	Booster dose	2.91 (0.43)	2.93 (0.43)	2.86 (0.47)	2.92 (0.46)	2.93 (0.50)	2.95 (0.51)	0.581	0.856	0.540
D-Dimer (ng/ml)	First dose	147.01 (122.09)	88.07 (93.30)	134.73 (110.2)	107.72 (114.95)	160.00 (125.7)	102.77 (190.93)	<0.0001	0.0005	<0.0001
	Second dose	147.73 (140.9)	155.22 (127.10)	142.51 (163.06)	136.87 (117.49)	144.65 (308.90)	131.74 (121.69)	0.004	0.039	0.002
	Booster dose	164.76 (123.12)	159.62 (121.51)	150.02 (123.29)	187.36 (382.92)	130.28 (113.04)	140.06 (135.56)	0.0002	0.0003	0.0362
Blood urea (mg/dL)	First dose	20.04 (5.70)	19.12 (4.91)	20.53 (5.97)	19.27 (4.90)	20.18 (6.32)	19.59 (4.63)	0.624	0.274	0.781
	Second dose	20.28 (5.90)	50.55 (5.94)	20.61 (6.13)	20.86 (6.44)	21.59 (6.63)	21.97 (6.99)	0.456	0.196	0.397
	Booster dose	19.26 (5.71)	19.12 (5.07)	19.06 (6.43)	19.50 (5.58)	19.48 (6.36)	19.45 (4.76)	0.183	0.296	0.001
BUN (mg/dL)	First dose	9.46 (2.68)	8.96 (2.35)	9.65 (2.86)	9.13 (2.37)	9.48 (3.00)	9.19 (2.28)	0.684	0.383	0.851
	Second dose	9.62 (2.77)	9.68 (3.11)	9.66 (2.90)	9.73 (3.00)	10.16 (3.18)	10.37 (3.37)	0.310	0.234	0.280
	Booster dose	9.01 (2.74)	8.89 (2.40)	8.95 (3.10)	9.10 (2.64)	9.30 (3.01)	9.13 (2.25)	0.183	0.182	0.001
Creatinine (mg/dL)	First dose	0.80 (0.15)	0.79 (0.19)	0.76 (0.19)	0.74 (0.12)	0.75 (0.15)	0.75 (0.15)	0.540	0.005	0.445
	Second dose	0.80 (0.16)	0.78 (0.15)	0.81 (0.16)	0.81 (0.16)	0.80 (0.19)	0.80 (0.16)	0.461	0.215	0.858
	Booster dose	0.75 (0.14)	0.73 (0.13)	0.77 (0.17)	0.77 (0.16)	0.77 (0.17)	0.76 (0.160)	0.223	0.479	0.643

A, Ashwagandha; B, Placebo; *p*, Mann Whitney/Chi-square, significance *p* < 0.05, Two tailed; All participants vaccinated with COVISHIELD^TM^ vaccination.

In this context, participants receiving Ashwagandha—particularly within the single-dose cohort—exhibited proportionally greater increases in SARS-CoV-2 S1- and RBD-specific IgG titres compared with placebo, with peak responses observed at weeks 12 for S1 and weeks 24 for RBD. Neutralizing antibody responses, assessed using sVNT and SNT, were maintained over the study period in both study groups. The safety and tolerability profile of Ashwagandha was remarkable. The NIH USA report had recommended a safe clinical use for 12 weeks (35). But for the first time, a safe use of Ashwagandha over 24 weeks has been demonstrated when administered under controlled conditions, providing a basis for future studies evaluating prolonged use in specific disorders (12, 13, 35).

Ashwagandha was administered in a dose of 500 mg once daily (equivalent to 4 g of standardized aqueous root extract per day) for prophylaxis use. The latter falls within the range (3–6 gm root powder) mentioned in a recent comprehensive safety report (36). The study participants were healthy and mostly young. The data collection included use of an indigenous mobile app (called Kawatch) in real time (24). A large repertoire of laboratory investigations was carried out. None of the participant withdrew (Figure 1) due to an AE. The AE in the current study were predominantly mild. Serious AEs were rare (0.08%) and not considered attributable to the study drug.

The study Ayurvedic physician attributed AE (none were serious) to Ashwagandha in 7 participants, and these were unexplained body weight gain, oral mucosal ulcers, acid-peptic symptoms, excessive sleep, and giddiness. The latter are well known in Ayurveda text and described in other studies (11, 35, 36). The incidence of gut related AE (such as dyspepsia, acid-peptic symptoms, diarrhea, constipation, acute abdomen) in the current study was less than 3% (95% CI 1.7, 4.3); nausea, anorexia, oral mucositis and altered taste < 1%. Mild abnormalities in the hepatic serum transaminases (1.5 times upper limit normal) were reported in 2% (95% CI 1.2, 3.5) of Ashwagandha and 0.7% (0.3, 1.7) placebo participants.

Overall, the safety findings observed in the present study are broadly aligned with the existing evidence base summarized in reports by the Ministry of Ayush and the report of NIH, which describe *predominantly short-term safety* of Ashwagandha when used within recommended doses. While the NIH highlights the need for caution in certain vulnerable populations, and notes limited evidence beyond shorter durations of use, the absence of serious adverse events over 24 weeks of administration in the present study provides additional empirical evidence supporting the safety of Ashwagandha over an extended, yet controlled, duration under clinical monitoring in the studied population. Similarly, some precautionary safety signals noted in reports from Denmark (DTU) were not observed in this investigation, further supporting the safe use of Ashwagandha in the studied population (35, 36).

In the current study, breakthrough SARS-CoV-2 infections were rare, mild, and uncomplicated, occurring in a very small proportion of vaccinated individuals with no significant difference between arms. These rates are comparable or less than previously reported incidences of breakthrough infections in vaccinated populations in India during earlier waves. During the second wave of the pandemic (2021), a longitudinal study reported a 16-weeks cumulative probability of breakthrough infection of 5.6% following primary vaccination, of which 81% received Covishield™ (33). Similarly, a large study among predominantly healthy young volunteers in medical institutions observed an 8.7% incidence of breakthrough infections within 14 days of primary vaccination (34).

The study was designed as an exploratory evaluation of prolonged Ashwagandha use for safety and potential immunomodulatory effects following vaccination. While initially intended to begin after the first primary Covishield™ dose, participants were also enrolled after the second dose or booster following a protocol amendment. Based on earlier reports, primary vaccination was expected to confer seroconversion in ~70–80% of the community (30, 32, 37–39), and a modest 5–7% increase in SARS-CoV-2-specific antibody titres in the Ashwagandha arm was hypothesized for sample size calculations. In practice, outcomes were influenced by unexpectedly high baseline and post-vaccination humoral responses (>90% seropositive for S1 and RBD IgG, predominantly neutralizing) and a substantial prevalence of nucleocapsid antibody positivity at baseline, reflecting prior exposure or infection. The kinetics and magnitude of the immune response to both natural SARS-CoV-2 infection and vaccination have been well characterized (40, 41). Nucleocapsid (NP) antibodies serve as a hallmark of prior symptomatic or asymptomatic SARS-CoV-2 infection and are not influenced by the vaccine platform used in this study.

This trial was conducted during, a critical phase in India's pandemic trajectory when SARS-CoV-2 transmission was widespread and asymptomatic/undocumented infection prevalence was exceptionally high. The unexpected finding that a good proportion of apparently healthy, RT-PCR–negative participants carried nucleocapsid antibodies at baseline reflects the real-world immunological milieu during this period—a phenomenon that was not anticipated initially and is now recognized as a major confounding factor.

The unexpected high baseline nucleocapsid seropositivity indicated widespread prior asymptomatic SARS-CoV-2 infection and, together with robust vaccine-induced immunity, resulted in a highly immunized cohort. This may have reduced the opportunity to detect any statistically significant humoral immunogenic effect of Ashwagandha. In addition, the study evaluated only humoral immune responses; therefore, potential effects on cellular immune pathways could not be assessed. Consequently, while no additional humoral immunogenic benefit of Ashwagandha could be discerned under the conditions of the present study, its effects on other components of the immune response remain to be investigated. It is possible that the immunogenic potential of Ashwagandha could be more appropriately evaluated earlier in the pandemic or immediately following vaccination. We speculate that the immunogenic potential of Ashwagandha may have been most evident immediately following the first vaccine dose (SDG), although the sample size in this subgroup was relatively small. Notably, the observed trend toward higher antibody titres with Ashwagandha in the SDG was not apparent in the DDG or BDG. All participants received Covishield™ (manufactured in India), a replication-defective chimpanzee adenovirus-vectored vaccine encoding the SARS-CoV-2 spike glycoprotein, which has been shown to elicit robust neutralizing antibody and cellular responses with unequivocal safety and efficacy (30, 37, 38).

Also, it should be noted that a combination of natural infection and specific vaccination was reported to be a powerful stimulus for protective immune response (44). In retrospect, taken altogether, we believe that the immunogenicity of Ashwagandha was indeed difficult to discern in the current context. Perhaps, the time framework of the current study should have been much earlier during the pandemic or perhaps soon after the vaccination was begun to evaluate the immunogenicity of Ashwagandha.

However, there were some trends in the specific antibody assay in the current study to favor Ashwagandha. The early and persistent humoral immune response (S1 and RBD) in the Ashwagandha arm was numerically superior, although not statistically significant, to the placebo in the participants enrolled soon after the first primary vaccination injection (SDG) (Table 2 and Table 3). This was also consistent with a better response in the virus neutralization assay (sVNT, Table 2). The immune response with Covishield™ is reported to increase after the second primary vaccination injection (30, 37). We speculate that there was a window of opportunity immediately after the first vaccine injection (SDG) to observe the immunogenicity of Ashwagandha. The sample size of the SDG was rather small Notably. This increment in the antibody assay with Ashwagandha in SDG was not seen in DDG or the BDG.

## 6 Strength and limitation

The current study was unique both from an Ayurvedic and COVID-19 vaccine research perspective. It was based on a plausible hypothesis that was supported by a large body of previous clinical and experimental data of Ashwagandha (described above). The study was designed during the pandemic and overlapped the vaccination drive in India. A controlled and prolonged administration of Ashwagandha for 24 weeks was novel. Although not the primary intention, the study provided important real-life data on the immune response (specific antibodies-S1, RBD and NP) to natural infection and Covishield™ vaccine in the Indian population. The good safety and tolerability to Ashwagandha in the current study seems a valid reference for future clinical research studies of Ashwagandha. The current study may be of relevance to the recently completed controlled drug trial (APRIL) of Ashwagandha in long COVID in UK; results not yet published (43). The study findings suggested that the Cohort immunity is not monolithic; baseline serological status profoundly influences the ability to detect incremental humoral antibody effects and clinical outcome of breakthrough infections.

There were several other limitations. Adherence to protocol and monitoring of study in young healthy participants was challenging and often cumbersome. As a consequence, 88.8% of participants were enrolled at a stage of mature or near-maximal vaccine-induced humoral response, where the ceiling effect substantially constrained the detectability of any adjunct immunomodulatory contribution. The SDG—the subgroup approximating early post-vaccination conditions most favorable for adjunct evaluation—comprised only 10.2% of the cohort and was not independently powered for subgroup inference. While the amendment was operationally necessary (detailed in Section 2.7), its immunological consequences are acknowledged as the primary methodological constraint of this trial.

The present study evaluated vaccine immunogenicity exclusively through humoral immune responses; cellular immune responses, including antigen-specific T-cell and innate immune function, were not assessed.

A higher daily dose of Ashwagandha may have been more effective, however the dose was decided based on clinical practice and guidelines of Ayurveda texts (36). We did not evaluate cellular immune response which seems important in case of Ashwagandha (19–21). We did not specifically assess other allegedly clinical benefits of Ashwagandha such as anxiety, depression and sleep that were relevant during the COVID pandemic (35, 42, 43). The results of the current study were limited to apparently healthy young individuals and may not be extrapolated to other population subsets such as the elderly and the immunosuppressed or those with other co-morbidity (including anxiety and stress disorders). However, considering the overall results (described above), the alleged immunogenicity of Ashwagandha in the current study should not be ruled out.

## 7 Conclusion

Standard oral daily administration of Ashwagandha (500 mg aqueous extract equivalent to 4 gm of root powder) over 24 weeks of prophylactic use was found remarkably safe and tolerable in this randomized placebo-controlled large sample size study in young participants. However, additional humoral immune response of Ashwagandha administration following Covishield™ vaccine could not be discerned. The vaccine response in the study population was unexpectedly high, and many study populations were found seropositive NC assay at baseline which indicated past asymptomatic SARS-CoV-2 infection. The incidence of breakthrough SARS-CoV-2 infections was low. More controlled clinical research is required to estimate the immunity benefits of Ashwagandha.

## Data Availability

The original contributions presented in the study are included in the article/Supplementary material, further inquiries can be directed to the corresponding author.
