# Supplementary material for: Safe prolonged prophylactic use and tolerability of Ashwagandha following COVISHIELD™ vaccination over 24 weeks: a multicentre randomized double-blind placebo-controlled trial
Source: Front Med (Lausanne). 2026 Aug 20;13:1852954. doi: 10.3389/fmed.2026.1852954 (PMC13539556; doi:10.3389/fmed.2026.1852954)
Supplement: Supplementary file 1 [file Data_Sheet_1.pdf]

Safe Prolonged Prophylactic Use and Tolerability of Ashwagandha Following COVISHIELD™ Vaccination Over 24 Weeks: A Multicentre Randomized Double-Blind Placebo-Controlled Trial

<u>Supplementary File: Quality Analysis of ingredients and tablets of *Withania somnifera*</u>

***Withania somnifera*-Root (Ashwagandha) (Raw drug)**

S. No	Physico chemical Parameters	Results	Specification	Remark
1.	Identification	Complies	Positive	Comply with In-house limits/ API limits
2.	Description	Complies	Primary root are straight , conical or finger like in shape, secondary root are thin and fibrous surface has longitudinal wrinkles	
3.	Color	Complies	Buff to grayish -yellow	
4.	Odor	Complies	Characteristic	
5.	Taste	Complies	Slightly bitter	
6.	Foreign matter	Complies	NMT2.00 %	
7.	Total alkaloid	0.048%	NLT 0.3%	
8.	Loss on drying at 105°	9.67%	NMT 12.00%	
9.	Total ash	4.77%	NMT 7.00%	
10.	Acid insoluble ash	0.83%	NMT1.00%	
11.	Alcohol soluble extractive	22.07%	NLT 15.00%	
12.	Heavy metals analysis			
	Lead	<0.5 ppm	<0.5 ppm	
	Mercury	<1 ppm	<1 ppm	
	Cadmium	<1 ppm	<1 ppm	
	Arsenic	<1 ppm	<1 ppm	

Ashwagandha, *Withania somnifera*-root (Aqueous Extract)

Sr. No.	Test Parameters	Results	Specification	Remarks
1.	Identification	Confirms	Characteristic to species	
2.	Appearance	Confirms	Fine Powder	
3.	Color	Confirms	Light to Dark Brown	
4.	Odor	Confirms	Characteristic	
5.	Taste	Confirms	Bitter	
6.	Particle Size	98.32%	NLT 90.00% through 80mesh	
7.	Bulk Density	0.35g/mL	0.30-0.70g/ml	
8.	Tap Density	0.50g/mL	0.40-0.80g/ml	
9.	Loss on Drying	3.90%	NMT 6.00%	
10.	Moisture Content	3.94%	NMT 4.00%	
11.	pH	5.70	5~8	
12.	Ash Content (Acid Insoluble)	1.90%	NMT 6.00%	
13.	Total Withanolides	0.30%	NLT 0.25% (as-is)	Comply with In-house limits/ API limits
14.	Herb Extract Ratio	Confirms	8:1	
15.	Extraction Solvent	Confirms	100% Water	
16.	Residual Solvents	Complies	USP <467> Screen	
17.	Test for Heavy Metals by AAS<br>Pb<br>Hg<br>As<br>Cd	 < 0.5ppm<br>< 0.1ppm<br>< 1ppm<br>< 0.3ppm	 < 0.5ppm<br>< 0.1ppm<br>< 1ppm<br>< 0.3ppm	
18.	Test for Microbiology(cft/gm)<br>Total Bacterial Count<br>Yeast &Mould Count	 <10cfu/g<br>Absent	 < 5000cfu/g<br>< 200cfu/g	
19.	Specific pathogens(gm)<br>Coliform<br>Enterobacteriaceae<br>*E.coli*<br>*Salmonella*<br>*S. aureus*	 Absent<br>Absent<br>Absent<br>Absent<br>Absent	 < 10cfu/g<br>< 100cfu/g<br>Absent/25g<br>Absent/25g<br>Absent/25g	
20.	Ingredients disclosure:	Specification/Requirement		
21.	*Withania somnifera* extract of root	NLT 98%		
22.	Maltodextrin	NMT 1% (Tackidex B167 (P00334) Roquette)		
23.	Silicon Dioxide	NMT 1% (Aerosil 200)		
24.	Shelf Life:	24months from the date of manufacture		
25.	Declaration:	This product has not been irradiated or ETO treated.		

Ashwagandha, *Withania somnifera*-Root (Extract Tablet)

Sr. No.	Test Parameters	Results	Specification	Remarks
1.	Description	Confirms	A red colour round shape, film coated tablet.	
2.	Color	Confirms	Red	
3.	Total Withanolides	0.14%	NLT 0.07%	
4.	Disintegration test	28 minutes 32 seconds	NMT 30 minutes	
5.	Weight variation	566.23mg	Average weight ±5% (513mg-567mg)	
6.	Hardness	7.1Kg/cm^2	NLT 3.0Kg/cm^2	
7.	Thickness	5.21mm	5.2 mm-5.7mm	
8.	Test for Heavy Metals by AAS<br>Pb<br>Hg<br>As<br>Cd	 <br>< 1ppm<br>< 1ppm<br>< 1ppm<br>< 1ppm	 <br>< 1ppm<br>< 1ppm<br>< 1ppm<br>< 1ppm	Comply with API limits
9.	Test for Microbiology(cft/gm)<br>Total Bacterial Count<br>Yeast &Mould Count	 <br>10cfu/g<br>Absent	 <br>< 10000cfu/g<br>< 200cfu/g	
10.	Specific pathogens(/gm)<br>Coliform<br>Enterobacteriaceae<br>*E.coli*<br>*Salmonella*<br>*S. aureus*	 Absent<br>Absent<br>Absent<br>Absent<br>Absent	 < 10cfu/g<br>< 100cfu/g<br>Absent/25g<br>Absent/25g<br>Absent/25g	
11.	Ingredients disclosure	Specification/Requirement		
12.	Excipients	Q.S		
13.	Colour	Erythrosine red		
14.	Packing	60 tablet in each HDPE white colour bottle		
15.	Shelf Life	36months from the date of manufacture		
16.	Storage	Store in cool and dry place, away from sunlight and moisture.		

Quantitative determination of total withanolides in *Withania somnifera*- Extract by HPLC

<Sample Information>

Sample Name	: WS4+WLA MIX STD
Sample ID	: WS4+WLA MIX STD
Data Filename	: STD-WS4+WLA--070719-013 lcd.lcd
Method Filename	: Withania-USP method.lcm
Batch Filename	: WITHANIA-070719-001.lcb
Vial #	: 1-2
Injection Volume	: 20 uL
Date Acquired	: 7/7/2019 8:48:53 PM
Date Processed	: 7/9/2019 3:29:00 PM

Acquired by	: HARDIK
Processed by	: HARDIK

<Chromatogram>

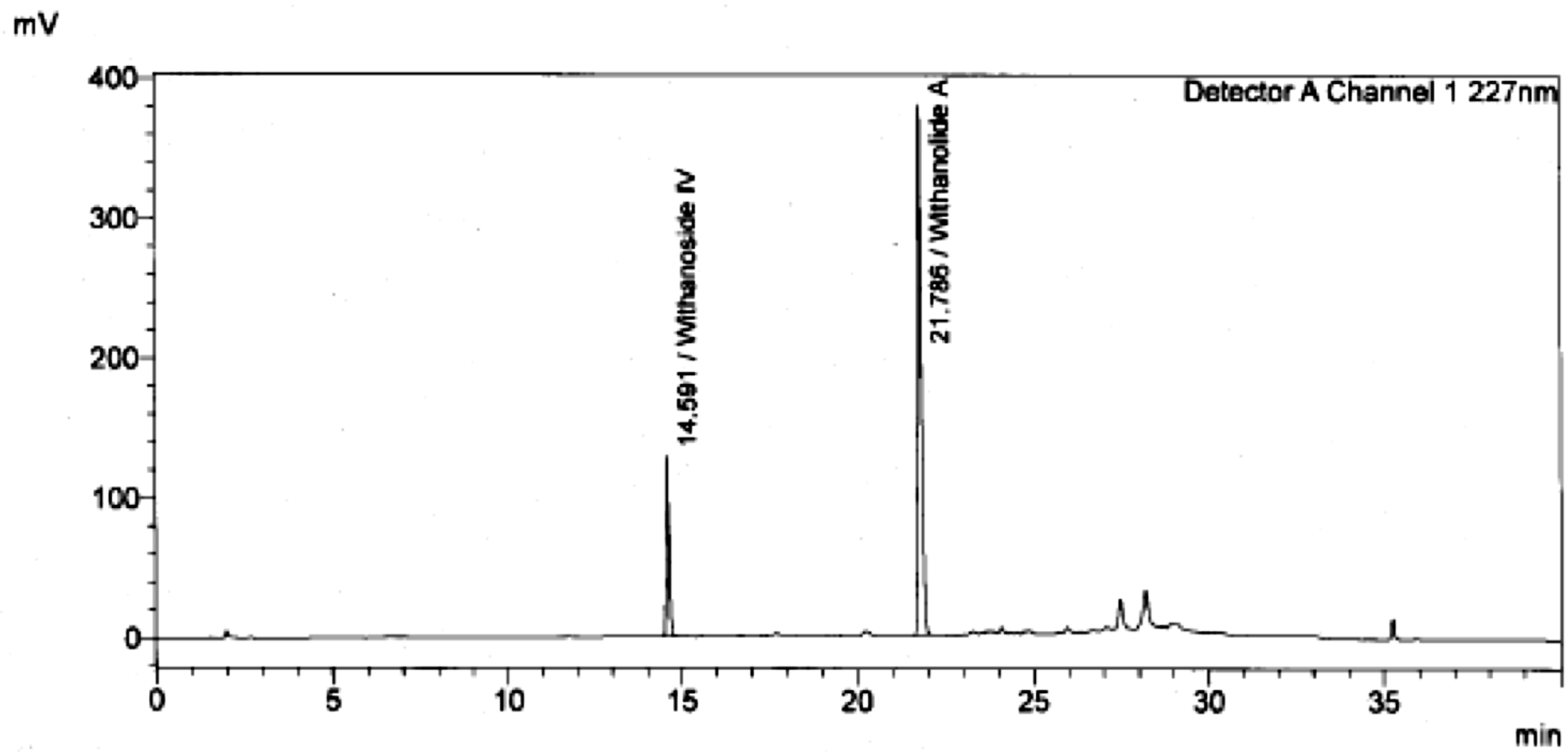

<Peak Table>

Detector A Channel 1 227nm

Peak#	Name	Ret. Time	Area	Height	Tailing Factor	Relative Retention Time
1	Withanoside IV	14.591	740320	129336	1.248	--
2	Withanolide A	21.786	2624362	378518	1.267	--
Total			3364682	507854		

<Sample Information>

Sample Name	: WITHANIA SOMNIFERA EXT
Sample ID	: 012619D0411WSEP-461KG
Data Filename	: PRD-WS-30000-080719-006.lcd
Method Filename	: Withania-USP method.lcm
Batch Filename	: WITHANIA-080719-001.lcb
Vial #	: 1-15
Injection Volume	: 20 uL
Date Acquired	: 7/8/2019 5:51:42 PM
Date Processed	: 7/9/2019 3:31:00 PM

Acquired by : HARDIK
Processed by : HARDIK

<Chromatogram>

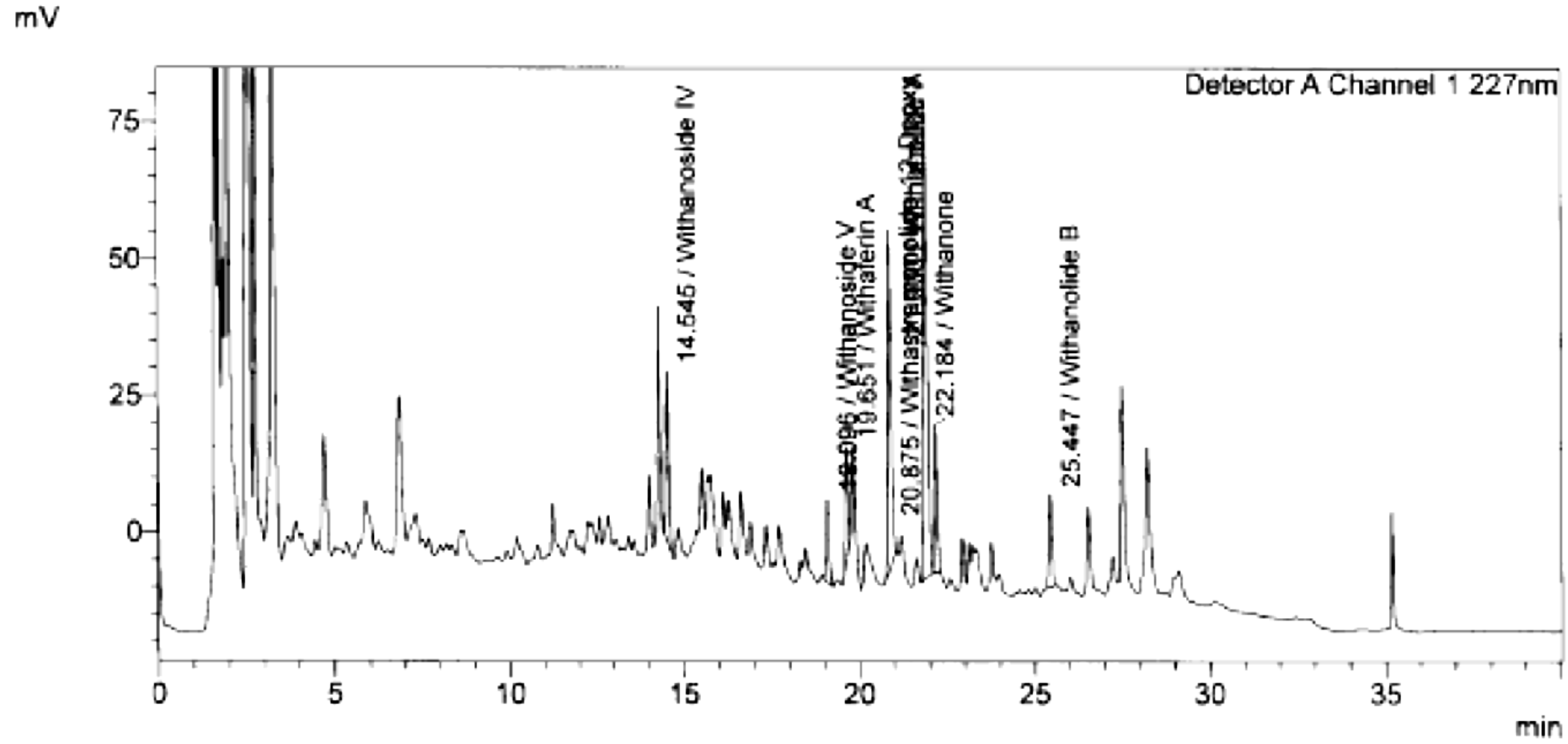

<Peak Table>

Detector A Channel 1 227nm

Peak#	Name	Ret. Time	Area	Height	Tailing Factor	Relative Ret. Time
1	Withanoside IV	14.545	183582	31208	1.215	0.664
2	Withanoside V	19.096	94419	15186	1.202	0.872
3	Withaferin A	19.651	120237	20574	0.982	0.897
4	Withastramonolide-12-Deoxy	20.875	441496	62030	1.196	0.953
5	Withanolide A	21.903	610218	87870	1.228	1.000
6	Withanone	22.184	153305	27120	1.296	1.013
7	Withanolide B	25.447	105633	16814	1.218	1.162
Total			1708891	260802		
