# Supplementary material for: Safe prolonged prophylactic use and tolerability of Ashwagandha following COVISHIELD™ vaccination over 24 weeks: a multicentre randomized double-blind placebo-controlled trial
Source: Front Med (Lausanne). 2026 Aug 20;13:1852954. doi: 10.3389/fmed.2026.1852954 (PMC13539556; doi:10.3389/fmed.2026.1852954)
Supplement: Supplementary file 2 [file Data_Sheet_2.docx]

**Supplementary Tables**

**Safe Prolonged Prophylactic Use and Tolerability of Ashwagandha Following COVISHIELD™ Vaccination Over 24 Weeks: A Multicentre Randomized Double-Blind Placebo-Controlled Trial**

**Supplementary Table S1: Table depicting the role of Ashwagandha on Quality of Life (WHOQOL)**

| Variable | **Dose** | **Baseline** | | **Mean (SD)**  **(Wk12)** | | **Mean (SD)**  **(Wk 24)** | | **Mean (SD)**  **(Wk 28)** | |
| --- | --- | --- | --- | --- | --- | --- | --- | --- | --- |
|  |  | **A** | **B** | **A** | **B** | **A** | **B** | **A** | **B** |
| Q1 | Single Dose  (n=94) | 3.73 (0.73) | 3.63 (0.69) | 3.96 (0.66) | 3.98 (0.66) | 3.89 (0.69) | 4.00 (0.71) | 3.89 (0.78) | 3.95 (0.65) |
|  | Double Dose  (n=383) | 3.74 (0.67) | 3.82 (0.68) | 3.87  (0.71) | 3.88 (0.71) | 3.80 (0.64) | 3.83 (0.65) | 3.84 (0.67) | 3.83 (0.69) |
|  | Booster Dose  (n=542) | 4.06 (0.60) | 3.99 (0.59) | 4.02 (0.60) | 3.99 (0.67) | 4.04 (0.67) | 4.01 (0.61) | 4.08 (0.66) | 4.11 (0.64) |
|  | Combined  (n=919) | 3.91 (0.66) | 3.89 (0.65) | 4.00 (0.61) | 3.94 (0.69) | 3.94 (0.67) | 3.95 (0.64) | 3.98 (0.69) | 3.99 (0.67) |
| Q2 | Single Dose  (n=94) | 3.82 (0.84) | 3.71  (0.81) | 3.96  (0.66) | 3.93 (0.77) | 3.98 0.57) | 4.0 (0.68) | 3.95 (0.83) | 3.93 (0.74) |
|  | Double Dose  (n=383) | 3.79 (0.66) | 3.79  (0.79) | 3.90 (0.67) | 3.91 (0.74) | 3.91 (0.66) | 3.85 (0.65) | 3.89 (0.73) | 3.84 (0.72) |
|  | Booster Dose  (n=542) | 3.93 (0.58) | 3.86 0.61) | 3.98 0.58) | 3.91 0.58) | 3.97 0.52) | 3.89 (0.61) | 3.99 (0.60) | 4.01 (0.68) |
|  | Combined  (n=919) | 3.87 (0.64) | 3.82 0.71) | 3.94(0.63) | 3.92 0.68) | 3.95 0.58) | 3.89 (0.63) | 3.95 (0.67) | 3.94 (0.70) |
| D1 | Single Dose  (n=94) | 25.65 (4.48) | 25.19(4.27) | 26.13 4.88) | 25.98 4.17) | 25.72(5.27) | 25.92 (4.24) | 26.02 (4.79) | 26.64 (4.16) |
|  | Double Dose  (n=383) | 26.15 (3.75) | 26.51 3.99) | 26.48(3.81) | 26.7 (3.85) | 26.73(3.72) | 26.83 (3.86) | 26.53 (4.03) | 26.90 (3.64) |
|  | Booster Dose  (n=542) | 26.83 (3.49) | 27.02(3.72) | 27.0 (3.19) | 27.08 3.49) | 26.69 3.44) | 26.88 (3.43) | 26.95 (3.18) | 27.35 (3.30) |
|  | Combined  (n=919) | 26.47 (3.69) | 26.65(3.91) | 26.70 3.69) | 26.79 3.75) | 26.60(3.77) | 26.76 (3.69) | 26.71 (3.68) | 27.11 (3.53) |
| D2 | Single Dose  (n=94) | 21.95 (3.92) | 22.04 3.54) | 22.34 3.49) | 22.00 3.10) | 21.66 3.67) | 22.15 (3.23) | 21.73 (3.61) | 22.16 (3.10) |
|  | Double Dose  (n=383) | 21.85 (2.30) | 21.72 3.06) | NA | 22.16 3.47) | 22.21(3.18) | 23.22(13.80) | 22.46 (3.16) | 22.34 (3.00) |
|  | Booster Dose  (n=542) | 22.39 (3.15) | 22.06 3.06) | 22.94 2.55) | 22.74 2.63) | 22.60 2.97) | 22.32 (2.76) | 22.82 (2.69) | 22.70 (2.99) |
|  | Combined  (n=919) | 22.15 (3.17) | 21.93 3.11) | 22.82 2.76) | 22.39(3.09) | 22.36(3.13) | 22.62 (8.52) | 22.59 (2.97) | 22.51 (3.00) |
| D3 | Single Dose  (n=94) | 10.02 (2.65) | 9.75 (2.95) | 10.77 3.43) | NA | 9.85 (3.04) | 9.71 (2.98) | 10.11 (2.72) | 10.02 (2.65) |
|  | Double Dose  (n=383) | 10.35 (2.61) | 10.05 2.67) | 10.41(2.93) | 10.08 2.73) | 10.80 2.88) | 10.43 (2.63) | 10.75 (2.71) | 10.35 (2.61) |
|  | Booster Dose  (n=542) | 9.91 (2.40) | 9.86 (3.10) | 9.80 (2.77) | 10.09 2.77) | 9.80 (2.55) | 9.83 (2.73) | 9.80 (2.19) | 9.91 (2.40) |
|  | Combined  (n=919) | 10.09 (2.51) | 9.92 (2.90) | 10.18 2.93) | 10.08(2.68) | 10.16 2.76) | 10.03 (2.74) | 10.17 (2.48) | 10.09 (2.51) |
| D4 | Single Dose  (n=94) | 28.52 (5.59) | 28.65 4.93) | 29.38 5.27) | 29.28 4.56) | 27.91 6.34) | 29.66 (4.63) | 28.80 (5.21) | 29.87 (4.51) |
|  | Double Dose  (n=383) | 29.08 (3.99) | 28.60 4.63) | 29.36 4.40) | 29.26 4.23) | 29.14(4.19) | 29.18 (3.96) | 29.38 (4.44) | 29.63 (4.16) |
|  | Booster Dose  (n=542) | 29.61 (4.37) | 29.11(4.30) | 29.89 4.35) | 29.92 4.31) | 29.82 3.85) | 29.23 (4.35) | 29.83 (3.54) | 29.66 (4.07) |
|  | Combined  (n=919) | 29.32 (4.36) | 28.87(4.49) | 29.61(4.48) | 29.55 4.31) | 29.38(4.32) | 29.38 (4.24) | 29.57 (4.06) | 29.67 (4.14) |
| The WHOQOL assessment included responses to individual questions (Q1 and Q2) and aggregated scores for the four domains: physical health (D1), psychological health (D2), social relationships (D3), and environment (D4). These scores were reported as means and standard deviations across four time points: baseline, week 12, week 24, and week 28, to evaluate changes in quality of life over time. *: p<0.05; **p<0.01 (Mann Whitney) | | | | | | | | | |

**Supplementary Table S2: Role of Ashwagandha administration on Quality of Life assessed through HRBHF scale**

| Dose | **Baseline** | | **Mean (SD)**  **(Wk12)** | | **Mean (SD)**  **(Wk 24)** | | **Mean (SD)**  **(Wk 28)** | | **p1** | **p2** | **p3** | **p4** |
| --- | --- | --- | --- | --- | --- | --- | --- | --- | --- | --- | --- | --- |
|  | **A** | **B** | **A** | **B** | **A** | **B** | **A** | **B** |  |  |  |  |
| Single Dose (n=103) | 673.55 (138.36) | 669.21 (154.55) | 711.14 (98.01) | 693.89 (124.54) | 713.29 (117.55) | 690.44 (107.16) | 691.55 (112.75) | 690.94  (105.06) | 0.97 | 0.76 | 0.19 | 0.93 |
| Double Dose (n=381) | 661.52 (121.56) | 658.72 (116.99) | 694.90  (109.29) | 699.51 (107.69) | 691.24 (104.34) | 692.29 (112.86) | 696.95 (114.87) | 695.22 (118.66) | 0.74 | 0.58 | 0.74 | 0.98 |
| Booster Dose (n=541) | 660.03 (104.77) | 664.84 (107.97) | 670.39 (108.53) | 658.02 (111.16) | 652.39 (112.02) | 654.68 (105.08) | 652.73 (110.64) | 652.14 (107.59) | 0.54 | 0.29 | 0.93 | 0.97 |
| Combined Dose (1025) | 661.87 (114.48) | 663.00 (116.84) | 686.02 (108.46) | 681.21 (112.91) | 673.12 (111.92) | 673.27 (109.73) | 673.62 (114.31) | 673.19 (113.59) | 0.80 | 0.72 | 0.96 | 0.99 |
| HR-BHF contained 9 questions pertaining to General health, anxiety, fatigue, energy, bowel habit, stress, happiness, sleep and appetite and participant response was marked for each question on a 100 mm visual analogue scale (anchored with best and worst response) to provide a total score ranging 0-900; n=number of participants, wk=week, p1,p2,p3p4 pertain to baseline, wk 12, wk 24, and wk 28 respectively, p5,p6, p7 pertain to mean change from baseline to week 12, week 24 and week 28 respectively | | | | | | | | | | | | |
